# The Whole is Other Than the Sum: Perceived Contrast Summation Within Color and Luminance Plaids

**DOI:** 10.1177/2041669516672481

**Published:** 2016-10-21

**Authors:** Avital S. Cherniawsky, Kathy T. Mullen

**Affiliations:** McGill Vision Research, Department of Ophthalmology, McGill University, Montreal, Quebec, Canada

**Keywords:** neuroscience, visual system, color vision, perception

## Abstract

The apparent contrast of a plaid is a reflection of the neural relationship between the responses to its two orthogonal component gratings. To investigate the perceived contrast summation of the responses to component gratings in plaids, we compared the apparent contrasts of monocular plaids to a component grating presented alone across chromaticity and spatial frequency. Observers performed a contrast-matching task for red–green color and luminance stimuli at low- and medium-spatial frequencies. Using the measured points of subjective equality between plaids and gratings, we evaluate perceived contrast summation across conditions, which may vary between 1 (no summation) and 2 (full summation). We show that achromatic plaids have higher perceived contrast summation than chromatic plaids. The greatest difference occurs at the medium-spatial frequency, with summation highest for achromatic plaids (1.87) and lowest for chromatic plaids (1.49), while at low-spatial frequencies, there is a smaller summation difference between achromatic (1.72) and chromatic (1.65) plaids. These results are consistent with recent theories of distinct cross-orientation suppression and summation mechanisms in color and luminance vision. Two control experiments for binocular versus monocular viewing, and the overall size of the stimulus patches did not reveal any differences from our main results.

## Introduction

Two-dimensional orthogonal gratings (plaids) are a useful tool in the study of complex form perception, as early spatial vision is well described by responses to simple one-dimensional sinusoidal gratings (Campbell & Robson, 1968; De Valois, Albrecht, & Thorell, 1982; Graham & Nachmias, 1971; Hubel & Wiesel, 1968; Marĉelja, 1980) that are combined at higher processing stages. The perception of more complex structures, such as plaids, is dependent on the processes of summation or suppression inherent to this combination. Furthermore, differences in the apparent contrast between color or achromatic plaids and a component grating presented alone may reflect characteristic summation and suppression processes. To evaluate perceived plaid and grating contrasts, we employed a contrast-matching paradigm in which the observer compares the contrast of a plaid to a single component grating. From these measurements, we describe the summation of responses to component gratings in the perceived contrast of plaids for different spatial frequency and chromaticity conditions. We evaluate the perceived contrast summation of suprathreshold orthogonal plaid components with respect to recent theories of distinct cross-orientation suppression and summation mechanisms in color and luminance vision.

In similar experiments, Georgeson and Shackleton (1994) and Tiippana, Näsänen, and Rovamo (1994) investigated the relative perceived contrasts of plaids and gratings for achromatic stimuli over a range of orientations and spatial frequencies with a contrast matching task using the method of adjustment. Both studies found that, when their Michelson contrasts were matched, two-dimensional plaids appeared to have lower contrast than single gratings across all spatial frequencies, that is, the perceived contrast of the plaid was less than a full summation of the combined plaid component contrasts. Georgeson and Shackleton noted that this effect increased with greater component orientation differences, which they attributed to a contrast normalization response across multiple orientation-tuned spatial frequency channels. Using a multiple-channel model, they found that the sublinear summation of component contrasts in plaid stimuli was well estimated by within-channel normalization of small signals to thresholds and quadratic summation across orientation-tuned channels.

There are most likely multiple sources of normalization in effect with suprathreshold cross-oriented stimuli, both at monocular subcortical sites (Baker, Meese, & Summers, 2007; Freeman, Durand, Kiper, & Carandini, 2002; Li, Peterson, Thompson, Duong, & Freeman, 2005; Sengpiel & Vorobyov, 2005; Truchard, Ohzawa, & Freeman, 2000; Walker, Ohzawa, & Freeman, 1998) and cortical orientation-tuned sites (Baker, Meese, & Summers, 2007; Heeger, 1992; Li et al., 2005; Meese & Holmes, 2007; Morrone, Burr, & Maffei, 1982; Sengpiel & Vorobyov, 2005; Walker et al., 1998). A well-known source of normalization for orthogonally presented grating stimuli is that of cross-orientation suppression, in which detection thresholds for a grating rise in the presence of a cross-oriented grating mask. Typically, for achromatic stimuli, cross-orientation suppression is highest under low-spatial and high-temporal frequency conditions, characteristic of a subcortical magnocellular pathway source (Burbeck & Kelly, 1981; Meese & Holmes, 2007). However, subsequent studies (Kim, Gheiratmand, & Mullen, 2013; Medina and Mullen, 2009) found that suppression in cross-orientation masking is actually greater for chromatic than achromatic stimuli across all spatiotemporal frequencies, indicating a role for the parvocellular pathway in the normalization of chromatic stimuli. Consequently, greater cross-orientation suppression may also be present at suprathreshold contrasts and reduce perceived contrast summation for chromatic plaids.

Physiological research in primates proposes two separate color sensitive systems based on different neural substrates with distinct spatial properties, one that integrates form and color and one that does not (Friedman, Zhou, & Heydt, 2003; Johnson, Hawken, & Shapley, 2001, 2008). While most color-sensitive neurons have the orientation-tuning required for edge and boundary detection, approximately 10% lack any orientation tuning with circular symmetric receptive fields better suited to the detection of colored surfaces and “blobs.” Recently, Gheiratmand, Meese, and Mullen (2013) and Gheiratmand and Mullen (2014) found analogous psychophysical evidence for these two types of response, based on measurements of subthreshold summation between orthogonal gratings. They observed increased subthreshold summation between orthogonal gratings specifically for low-spatial frequency red–green color vision, indicative of detection mechanisms without orientation tuning (isotropic). Increased summation was also observed for binocular summation between dichoptic low-spatial frequency color gratings, but, unlike monocular vision, this summation mechanism is most likely orientation tuned (Gheiratmand, Cherniawsky, & Mullen, 2016). We, therefore, ask whether correspondingly greater cross-orientation summation, which elevates contrast sensitivity and can be characteristic of an isotropic mechanism, is also evident at suprathreshold contrasts for low-spatial frequency color stimuli.

In this article, we compare the apparent contrast of monocular plaids to a component grating for both red–green chromatic and achromatic contrast stimuli, and at low- and medium-spatial frequencies. With this method, we may demonstrate the differing effects of suppression (Kim et al., 2013; 2013; Medina & Mullen, 2009) and summation (Gheiratmand et al., 2013; Gheiratmand & Mullen, 2014) specific to colour vision as compared with achromatic vision. The two reviewed color-specific cross-orientation effects, suppression and summation, have different spatial frequency signatures, as increased suppression affects all spatial frequencies, whereas increased summation is only present at low-spatial frequencies. These effects are typically observed in contrast sensitivity, but they may also affect higher contrast perception. We also complete two separate control experiments to test the effects of stimulus size and binocular presentation. The first control experiment addresses a potential confound of stimulus size by equating the number of spatial grating cycles displayed between spatial frequency conditions. The second control experiment repeats the contrast matching task with binocular stimuli to examine any potential differences between monocular and binocular presentation.

## Methods

### Apparatus

All stimuli were displayed with a ViSaGe video-graphics card (Cambridge Research Systems, Kent, UK, 14-bit resolution). Stimulus presentation was on a cathode ray tube (CRT) computer monitor with a resolution of 1024 × 768 and refresh rate of 120 Hz (Iiyama Vision Master Pro 513, Iiyama Corporation). The monitor was gamma corrected with a ViSaGe program and OptiCal photometer from Cambridge Research Systems and red, green, and blue phosphor displays were calibrated using a PR-645 Spectrascan spectroradiometer (Photo Research Inc., Chatsworth, CA, USA). CIE 1931 *x–y* chromaticity coordinates were red: *x* = 0.628, *y* = 0.344, green: *x* = 0.283, *y* = 0.613, and blue: *x* = 0.151, *y* = 0.073. The background was achromatic with a mean luminance of 43 cd/m^2^. All stimuli viewing in monocular conditions were done with a custom-built, modified 8-mirror Wheatstone stereoscope with the untested eye observing the mean luminance background. The observer was 58 cm from the screen center. Binocular viewing conditions in the control experiment were done without the stereoscope with the observer at the same distance from the screen.

### Observers

There were eight observers, one author (A. S. C.) and the rest naïve. Of these observers, three (A. S. C., A. F., and J. T.) also completed a binocular control. Two observers (C. C. and J. T.) also completed a control experiment for stimulus size. All participants had normal or corrected-to-normal visual acuity and normal color vision, as assessed with the Farnsworth-Munsell 100 Hue test. The experiment was performed in accordance with the Declaration of Helsinki and approved by the institutional ethics committee of McGill University Health Centre. All participants signed an informed consent form.

### Stimuli

**Figure 1.**
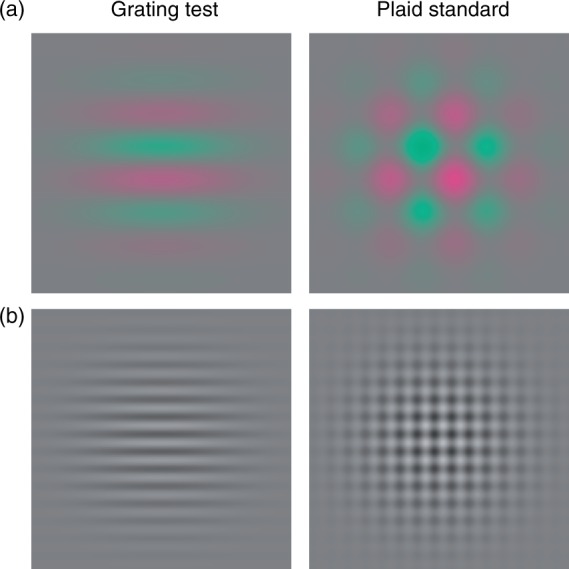
Example variable grating and standard plaid stimuli for 0.375 c/deg red-green isoluminant (a) and 1.5 c/deg achromatic (b) conditions. In a contrast matching task, observers were asked to indicate which of these stimuli appeared stronger in contrast as the grating stimulus varied in contrast across trials.

Figure 1 shows example stimuli. Stimuli were isoluminant red–green or achromatic sine-wave gratings with a phase of 0°, either presented alone as horizontal gratings or combined with an identical vertical grating to form a plaid. Two spatial frequencies were used (low: 0.375 c/deg or medium: 1.5 c/deg) displayed in a 10° circular patch, fixated centrally. A control experiment was also done in which the medium-spatial frequency stimuli was reduced in size and displayed in a 2.5° circular patch so that it had the same number of spatial cycles as the low-spatial frequency. Stimulus edges were contrast enveloped with a spatial raised cosine of 2.5°. All stimuli were static and presented within a contrast modulated temporal Gaussian envelope with a sigma of 0.125 s, in a stimulus window of overall duration of 0.5 s, with an interstimulus interval of 0.4 s. For plaid stimuli, the two components were created on the screen in separate frames and combined by interleaving on alternate frames through the ViSaGe. For grating stimuli, grating and a mean luminance frame were interleaved. This presentation method was kept consistent across all experiments, including threshold detection. Component grating contrast is reported in all figures.

### Color Space

The grating contrast is defined within a three-dimensional cone-contrast space (Cole, Hine, & McIlhagga, 1993; Sankeralli & Mullen, 1996). For details on the calculation of this cone-contrast space, please refer to Gheiratmand and Mullen (2014). Grating contrast is defined by the vector length in cone contrast units (CC):

**Figure:**
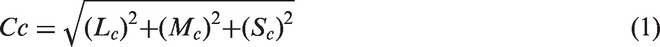

where *L_c_*, *M_c_*, and *S_c_* represent the L, M, and S Weber cone-contrast fractions in relation to the L, M, and S cone values of the achromatic background. (This corresponds to Michelson contrast scaled by

**Figure:**
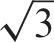

.) Each observer had their spatial frequency specific red–green isoluminance point measured using a standard minimum motion task (Gheiratmand & Mullen, 2014).

### Protocols

The main experiment was done monocularly with the right eye and took place over multiple sessions. There were four stimuli conditions (2 × 2): Chromaticity × Spatial frequency. For each condition, contrast detection thresholds were determined for an orthogonal plaid, the standard stimuli in the matching experiment. Thresholds were obtained using a two-alternative-forced choice method of constant stimuli as described previously (Gheiratmand & Mullen, 2014). Psychometric functions had six or more contrast levels with 60 to 80 trials per level. Multiples of plaid detection thresholds, measured for each chromaticity and spatial frequency were used to equate between conditions.

For the contrast-matching paradigm, a two-alternative-forced choice method was employed in which one interval contained the standard plaid stimuli set to three times threshold contrast and the other interval contained a single test grating stimuli which varied between trials along six contrast levels (step size of 2.5 dB). Observers were asked to determine which stimulus appeared stronger in contrast and to indicate their answer with a button press. No feedback was given for this subjective task. Grating test contrast ranges were adjusted for each block so that resulting psychometric functions included equivalent numbers of test contrasts that were perceived to be either stronger or weaker than the given standard plaid contrast. There were also two control experiments that were completed after the main experiments. For three participants, the experiment was repeated with binocular stimuli shown without the stereoscope. Two participants also repeated the monocular, medium-spatial frequency conditions with a smaller stimulus size that had the same number of cycles (3.75) as the low-spatial frequency stimuli. The point of subject equality (PSE) for grating test contrasts was determined for each condition. PSEs are the 50% point on the psychometric function and were based on 80 to 100 trials per contrast level. Unlike Georgeson and Shackleton (1994), we report plaid contrasts as the contrast of one plaid component, not as the sum of both components:

**Figure:**
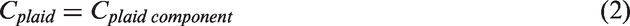

This plaid contrast calculation was chosen so as to be consistent with plaid contrasts in previous subthreshold summation experiments (Gheiratmand et al., 2013, 2016; Gheiratmand & Mullen, 2014).

### Analysis

We are interested in the relationship between the apparent contrast of a grating and standard plaid because it should reveal the degree of summation between the two components of the plaid. If the plaid’s contrast is perceived as the full sum of its components, an observer will judge a grating as equal to the plaid standard when the grating is set to twice the physical contrast of one of the plaid component gratings. In a similar manner to the calculation of summation ratios (Gheiratmand & Mullen, 2014; Meese & Baker, 2011; Watson, 1982), we quantified the amount of perceived contrast summation as the ratio of the PSE contrast of the test grating (*PSE_Grat_*) to the physical contrast of the standard plaid (*C_plaid_*):

**Figure:**
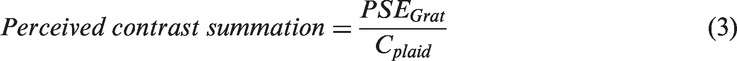

If perceived contrast summation is 2, as in the previous example, there is a full summation of perceived plaid component contrasts in the plaid. However, if summation is 1, observers perceived the stimuli as equal when a grating has the same physical contrast as just one component of the plaid, and there is no perceived contrast summation between plaid component contrasts in the plaid stimuli.

## Results

**Figure 2.**
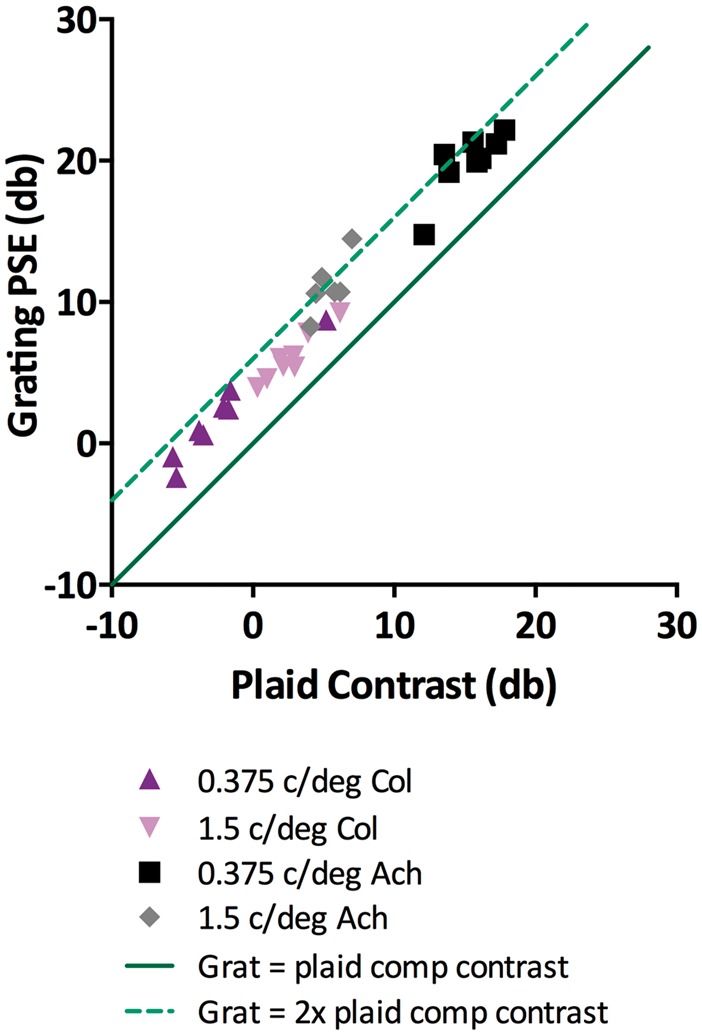
Individual observer (*n* = 8) points of subjective equality (PSE) for test gratings are plotted against standard plaid contrast in dB, where 1% contrast is 0 db. Low-spatial frequency (0.375 c/deg) color condition points are upright purple triangles, medium-spatial frequency (1.5 c/deg) color are inverted pink triangles, 0.375 c/deg achromatic are black squares, and 1.5 c/deg achromatic are gray diamonds. For ease of interpretation, a dark green line is plotted when grating contrasts equal one plaid component contrast and a light green dashed line when grating contrasts equal both (2×) plaid component contrast.

Figure 2 shows results from the main contrast matching experiment for individual observers. The PSEs of test gratings are plotted with respect to their matched standard plaid contrasts, which were presented at three times detection threshold. In all cases, error bars were smaller than the plotted symbols, as contrast matching tasks typically have low within-subject variability (Baker, Meese, & Georgeson, 2007; Tiippana, Rovamo, Näsänen, Whitaker, & Mäkelä, 2000). The solid line in Figure 2 represents points for which grating contrast equals plaid component contrast, and the dashed line represents grating contrast at twice the plaid component contrast (+6 dB). As detection thresholds vary significantly with chromaticity and spatial frequency (Mullen, 1985), matching was done across a wide range of contrasts depending on condition and observer. Overall, grating PSEs fell between one and two times the plaid contrast, as was expected. However, some individuals had achromatic PSEs that were more than twice the standard plaid contrast (above the light green dashed line). While average achromatic summation was below 2, these individual results represent the upper range. Previous achromatic contrast matching experiments have found that plaid contrasts appear lower than their combined component contrasts (below the light green dashed line on our plot; Georgeson & Shackleton, 1994; Tiippana et al., 1994).

**Figure 3.**
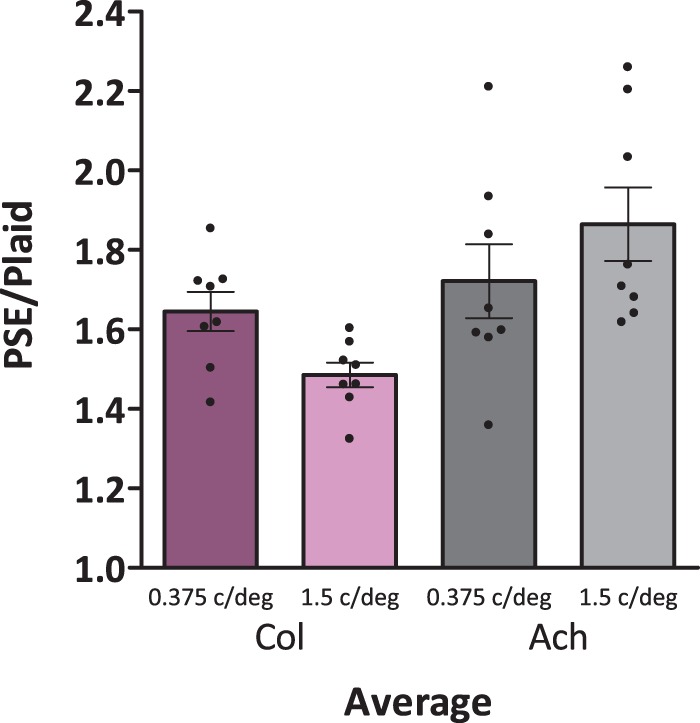
Average (bars) and individual (dots) perceived contrast summation is plotted as the PSE of a test grating divided by the contrast of the standard plaid. If perceived contrast summation is 2, then twice the amount of grating contrast is required to be perceived as equal to a plaid, while if perceived contrast summation is 1, plaids are perceived to be equal to a single component grating contrast. A purple bar is used for the low-spatial frequency (0.375 c/deg) color condition, pink for medium-spatial frequency (1.5 c/deg) color, dark gray for 0.375 c/deg achromatic, and light gray for 1.5 c/deg achromatic.

**Figure S1.**
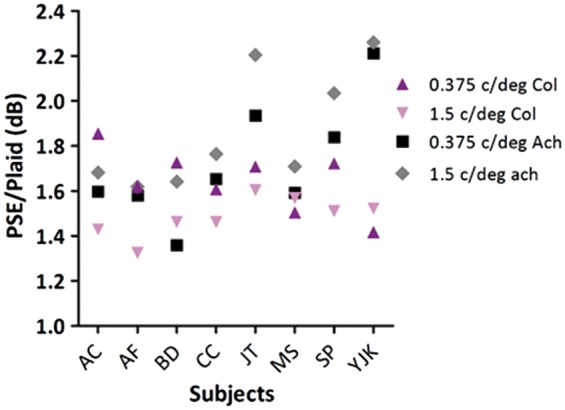
Individual perceived contrast summation is plotted as the PSE of a test grating divided by the contrast of the standard plaid. Low spatial frequency (0.375 c/deg) colour condition points are upright purple triangles, medium spatial frequency (1.5 c/deg) colour are inverted pink triangles, 0.375 c/deg achromatic are black squares, and 1.5 c/deg achromatic are grey diamonds.

Average and individual perceived contrast summation ratios are plotted in Figure 3 (individual ratios are further detailed in Supplementary Figure S1). A ratio of 1, corresponding to the dark green solid line in Figure 2, would indicate that observers perceived plaids and gratings as equal in contrast when the plaid component contrast equaled the grating contrast, while a ratio of 2, corresponding to the lighter green dashed line in Figure 2, would indicate that observers perceived plaids and gratings as equal in contrast when the sum of the two plaid component contrasts equaled the grating contrast. A ratio of 1 therefore indicates no summation or inhibition between component gratings, and 2 indicates full summation. Average perceived contrast summation ratios for all eight subjects with *SEM* are as follows: 1.65 ± 0.05 for low-spatial frequency color plaids, 1.49 ± 0.03 for mid-spatial frequency color plaids, 1.72 ± 0.09 for low-spatial frequency achromatic plaids, and 1.87 ± 0.09 for mid-spatial frequency achromatic plaids. An estimated perceived contrast summation ratio for Georgeson and Shackleton’s (1994) results in a similar experiment with 1 c/deg achromatic stimuli is around 1.5, which is lower than our achromatic ratios. This difference may be due to natural sampling variation or differences in methodology.

A repeated measures ANOVA of perceived contrast summation ratios with two factors (2 Chromaticity × 2 Spatial frequency) reveals a significant main effect of chromaticity, *F*(1,7) = 5.619, *p* = .050, η*_p_*^2 ^= .445, with no main effect of spatial frequency, *F*(1,7) = 0.056, *p* = .820, η*_p_*^2 ^= .008, and a significant interaction between the two conditions, *F*(1,7) = 16.389, *p* = .005, η*_p_*^2 ^= 0.701. Overall achromatic ratios were higher than chromatic. Finding a significant interaction, we also did a pair-wise *t* test with a Holm-Bonferroni correction between spatial frequencies for each chromatic condition. Perceived contrast summation was greater for low than medium-spatial frequencies for chromatic stimuli, *t*(7) = 2.507, *p* = .041 (α = 0.05) and was greater for medium than low-spatial frequencies for achromatic stimuli, *t*(7) = 4.248, *p* = .004 (α = 0.025).

**Figure 4.**
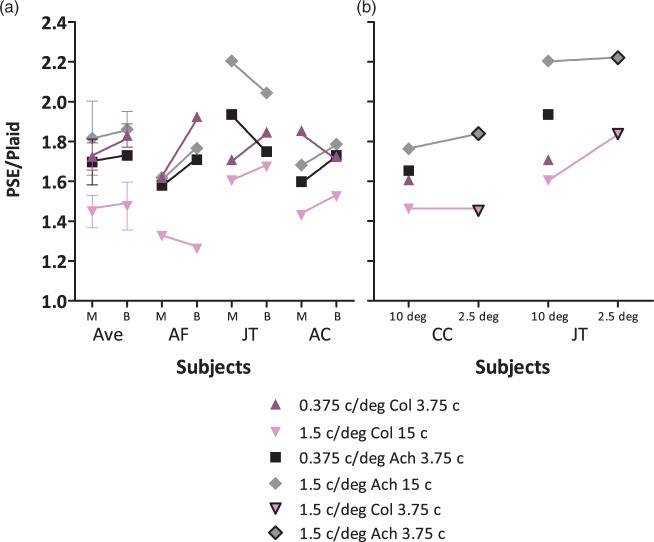
Perceived contrast summation is plotted for the two control experiments. Individual perceived contrast summation from the original and control experiments are plotted next to each other with a colored line connecting repeated conditions. For the binocular control experiment (a), “M” denotes the original monocular experiment and “B” the binocular control. For the smaller stimulus size control experiment (b), “10deg” refers to the stimulus size in the original experiment and “2.5deg” refers to stimulus size in the control experiment where the 1.5 c/deg conditions were repeated to have an equal number of grating cycles (3.75) to the low-spatial frequency (0.375 c/deg) condition. 0.375 c/deg color condition points are upright purple triangles, medium-spatial frequency (1.5 c/deg) color are inverted pink triangles, 0.375 c/deg achromatic are black squares, 1.5 c/deg achromatic are gray diamonds, and 1.5 c/deg stimuli with 3.75 instead of 15 cycles are outlined in black.

Three observers also completed a binocular control experiment. These perceived contrast summation results, compared with their monocular data, are presented in Figure 4(a). While the variability is quite high, there was no systematic change in perceived contrast summation between monocular and binocular conditions. A repeated measures ANOVA of perceived contrast summation ratios on these three observers with three factors (2 Chromaticity × 2 Spatial frequency × 2 Ocularity) found no effects of monocular or binocular presentation, *F*(1,2) = 1.046, *p* = .414, η*_p_*^2 ^= .343. Additionally, there were no interactions of ocularity with chromaticity, *F*(1,2) = 0.112, *p* = .770, η*_p_*^2 ^= .053, spatial frequency, *F*(1,2) = 0.168, *p* = .721, η*_p_*^2 ^= 0.078, or all three, *F*(1,2) = 0.172, *p* = .719, η*_p_*^2 ^= .079. Two observers also completed a second control condition, presented in Figure 4(b), in which they repeated the mid-spatial frequency conditions with smaller stimuli, 2.5°, and fewer cycles, 3.75, which equated the number of cycles with the low-spatial frequency conditions. If mid-spatial frequency plaids appear higher in contrast due to more plaid conjunctions, reducing its size and number of conjunctions should negate this effect. However, for these observers, mid-spatial frequency perceived contrast summation ratios did not decrease with a smaller grating cycle number.

## Discussion

### Plaids are Perceived as Other Than the Sum of Their Components

As in previous research (Georgeson & Shackleton, 1994; Tiippana et al., 1994), we find that observers perceive plaids to have lower contrasts than the combined contrasts of their plaid components, that is, there is less than full perceived contrast summation for all conditions. Significantly, we also find that perceived contrast summation differed with spatial frequency and chromaticity, suggesting different levels of contrast suppression or summation depending on stimulus conditions. Overall, chromatic plaids have lower perceived contrast summation than achromatic plaids. Moreover, this effect is greater for mid-spatial frequency stimuli; for this spatial frequency, chromatic plaids have the lowest perceived contrast summation while achromatic plaids have the highest. Recent research on cross-orientation effects in color vision has identified two different and specific mechanisms: subthreshold summation, which acts to increase contrast sensitivity to cross-oriented stimuli (Gheiratmand et al., 2013, 2016; Gheiratmand & Mullen, 2014), and cross-orientation suppression, which decreases contrast sensitivity when one cross-oriented component is at a suprathreshold contrast (Kim et al., 2013; Medina & Mullen, 2009). We next discuss how the presence of these mechanisms in suprathreshold plaid contrast perception is supported by our data.

### Greater Cross-Orientation Suppression in Color Plaids

Lower perceived contrast summation for chromatic plaids may be due to stronger cross-orientation suppression in color vision, which has been found to operate across all spatial frequencies for both monocular and binocular stimuli (Kim et al., 2013; Medina & Mullen, 2009). While achromatic suppression may be due to subcortical magnocellular sources, monocular chromatic cross-orientation suppression is likely found in the cortex (Kim et al., 2013; Solomon & Lennie, 2005). The lower perceived contrast summation for chromatic compared with achromatic plaids that we find complements these previous findings of increased cross-orientation suppression with both monocular and binocular stimuli. The stimuli in this experiment were presented both monocularly and binocularly (in a control condition) with no discernable differences between conditions.

### Increased Summation for Low-Spatial Frequency Color Plaids

We find that although the color plaids have lower overall perceived contrast summation than achromatic plaids, summation is noticeably higher for the low compared with the medium-spatial frequency chromatic plaids. As previous research has found cross-orientation suppression to be similar for both low- and medium-spatial frequency chromatic plaids (Kim et al., 2013; Medina & Mullen, 2009), it is unlikely that differences in cross-orientation suppression account for this spatial frequency effect. Alternatively, low-spatial frequency color plaids may benefit from greater contrast summation, increasing perceived contrast relative to the medium-spatial frequency. In the Introduction section, we note that there is both psychophysical and physiological evidence for monocular isotropic color detection mechanisms at low-spatial frequencies (De Valois, 1965; Friedman et al., 2003; Gheiratmand et al., 2013, 2014; Johnson et al., 2001, 2008). Although it is unlikely that an isotropic response is acting in isolation at suprathreshold contrasts, color-sensitive isotropic cells could be part of a larger population response to suprathreshold color plaids at low-spatial frequencies, increasing cross-orientation contrast summation. Potentially, while greater cross-orientation suppression reduces chromatic plaid contrast summation compared with achromatic plaids, an isotropic mechanism may still boost summation for low- over medium-spatial frequency chromatic plaids.

### A Comparison to Colour and Luminance Effects

A recent study by Kim and Mullen (2016) conducted a contrast-matching experiment using plaids composed of color and luminance contrast to investigate the effect of color contrast on luminance contrast and vice versa. The combined color or luminance plaids were compared with color or luminance gratings to assess the perceived contrast of each plaid component in the presence of the other. A comparison can be made between our results and theirs for monocular presentation at similar spatiotemporal frequencies. Kim and Mullen (2016) found that the perception of color contrast was increased in a color or luminance plaid more so than the perception of luminance contrast, that is, color contrast benefits from the addition of a cross-oriented luminance grating but not vice versa. While the perception of color contrast was facilitated by luminance contrast, perceived contrast summation was still less than that found for our color or color plaids. Averaged over spatial frequency, color contrast was enhanced by 32% (a summation ratio of 1.32) in the presence of cross-oriented luminance contrast, while, in our study, color contrast was enhanced by 57% (1.57) in the presence of cross-oriented color contrast. Thus, perhaps not surprisingly, summation between color contrast components is greater than across components of different contrast types.

### Increased Summation for Medium-Spatial Frequency Achromatic Plaids

Revealing an opposite spatial frequency effect to color plaids, medium-spatial frequency achromatic plaids have greater perceived contrast summation than low-spatial frequency achromatic plaids. This effect is unexpected and, to our knowledge, a similar result has not been reported before. For achromatic stimuli, increased cross-orientation suppression is found at low-spatial and high-temporal frequencies but is insignificant at low temporal frequencies (Burbeck & Kelly, 1981; Meese & Baker, 2011). As our stimuli are static, it is unlikely that increased cross-orientation suppression is reducing perceived contrast summation at the low-spatial frequency. However, cross-orientation suppression is not the only source of normalization that may be reducing contrast perception for our plaid stimuli, but with our limited number of data points, we cannot employ a model that could effectively estimate normalizations mechanisms including cross-orientation or self-suppressive effects.

### Contrast Constancy Across Stimulus Size

There is some debate as to whether the visual processing of plaids make use of “conjunction detectors” (Peirce, 2007, 2011) or the same detectors that are used for their component gratings (May & Zhaoping, 2011, 2013). If there are plaid conjunction detectors, then it is possible that a greater number of conjunctions could result in an increased contrast perception of plaids, compared with their components. As our original experiment equated stimulus size, there were more plaid conjunctions present in the medium-over the low-spatial frequency. This potential confound could therefore increase perceived contrast summation at the medium-spatial frequency and potentially explain the observed spatial frequency effect in achromatic stimuli. However, a control experiment found that smaller medium-spatial frequency stimuli, with fewer plaid conjunctions, did not have lower contrast summation. While there is evidence for contrast constancy with respect to stimulus area or number of cycles in the perceived contrast of achromatic (Cannon & Fullenkamp, 1988; Takahashi & Ejima, 1984) and chromatic gratings (Tiippana et al., 2000), there is little research on the contrast constancy of plaids compared with gratings. A small experiment with one subject was included in the Georgeson & Shackleton (1994) study in which they completed a plaid and grating contrast matching task across different plaid component orientation differences at two stimuli sizes (2 and 5 deg). They also found that there was little difference between the two contrast matching functions, indicating that the perceived contrast of plaids and gratings may be equally affected by stimulus size.

### Conclusion

We find that the contrast perception of complex forms and their components depends on spatial frequency and chromaticity. In sum, relative to component gratings, color plaids have lower perceived contrast summation than achromatic plaids and this chromatic summation difference is greater at the medium-spatial frequency and smaller at the low-spatial frequency. These results may be reflective of two separate processes: greater cross-orientation suppression in color vision (Kim et al., 2013; Medina & Mullen, 2009) and increased cross-orientation summation for low compared with medium-spatial frequency color stimuli (Gheiratmand et al., 2013, 2016; Gheiratmand & Mullen, 2014).

## Appendix

Figure S1 Perceived contrast summation for individual subjects
